# Progress of Research on the Metabolic Regulation of Lactylation in Muscle Tissues and Its Disease Associations

**DOI:** 10.3390/biom16020212

**Published:** 2026-01-30

**Authors:** Zhihang Wang, Ji Zhang, Junxi Wu, Guangrun Liu, Yun He, Hongbo Zhao, Xiaolin Jiang, Shengbo Yang

**Affiliations:** Department of Anatomy, Zunyi Medical University, Zunyi 563099, China; wangzhihang@zmu.edu.cn (Z.W.); zhangji@zmu.edu.cn (J.Z.); wujunxi@zmu.edu.cn (J.W.); liuguangrun@zmu.edu.cn (G.L.); heyun@zmu.edu.cn (Y.H.); zhaohongbo@zmu.edu.cn (H.Z.); jiangxiaolin@zmu.edu.cn (X.J.)

**Keywords:** muscle tissue, lactylation, metabolic reprogramming, muscular diseases, epigenetics

## Abstract

Lactylation serves as a vital link between cellular metabolism and epigenetic regulation and plays a pivotal role in muscle biology. Muscle tissue is the primary site of lactate production; its unique metabolic environment confers dynamism, specificity and functional diversity for lactylation. Under physiological conditions, lactylation regulates myocyte energy metabolism, proliferation, differentiation, and exercise adaptation through a dynamic “writer–eraser–reader” mechanism. In pathological states, lactate imbalance directly contributes to the progression of various muscular disorders. For instance, diminished histone lactylation during muscle aging suppresses the expression of genes critical for DNA repair and protein homeostasis. Aberrant lactylation is involved in the development of insulin resistance and diabetic cardiomyopathy. Furthermore, lactylation exerts dual effects in cardiovascular diseases; it provides protection by enhancing the transcription of repair genes and simultaneously aggravates injury by promoting processes such as fibrosis and ferroptosis. Collectively, these findings underscore the importance of lactylation in muscular pathologies and provide a theoretical foundation for the development of therapies that target this modification process. As the regulatory mechanisms of lactylation have become clearer, precise interventions targeting specific modification sites are expected to open new therapeutic avenues for muscular diseases.

## 1. Introduction

Lactate has long been recognized as both a metabolic byproduct and energy substrate, and recent studies have revealed that it serves as a substrate for lactylation, thereby contributing to epigenetic regulation [1]. This pivotal finding has established a new research frontier linking metabolic processes to epigenetic mechanisms [2].

Under physiological conditions, skeletal muscle is the site of approximately 25% of the total body lactate production, which increases to 70% during intense exercise [3,4]. Although cardiac muscle relies predominantly on aerobic metabolism, lactate production can increase significantly under hypoxic conditions. The production rate rises from 1.22 μmol·g dry weight^−1^·min^−1^ at rest to 18.5 μmol·g dry weight^−1^·min^−1^ [5]. Similarly, smooth muscle can produce lactate via anaerobic metabolism during hypoxia or vigorous intense contraction, with its content increasing from 29.83 ± 5.05 mg/100 g of wet weight to 65.36 ± 7.37 mg/100 g of wet tissue [6]. Lactate can serve as a substrate for protein lactylation, thereby participating in the regulation of cellular function.

Protein lactylation in muscle tissue exhibits four key characteristics: (1) Dynamic nature: lactylation levels change rapidly in response to muscle activity [7]; (2) Specificity: distinct modification patterns across different muscle fiber types [8]; (3) Functional significance: regulatory role in diverse physiological processes from energy metabolism to gene expression [9,10]; (4) Pathological relevance: dysregulated lactylation is associated with multiple muscular disorders, including muscle aging, metabolic myopathies, and cardiovascular diseases [11,12,13].

This review focuses on the metabolic regulatory mechanisms of lactylation in muscle tissues and their associations with disease, aiming to establish a foundation for further investigation into muscle pathophysiology and the development of therapeutic interventions.

## 2. Discovery and Characterization of Lactylation

The discovery of protein lactylation represents the progression of research from initial observations to developing a mechanistic understanding. In the late 1990s, lactyl-N-acetylneuraminic acid was identified in the mandibular glands of horses and human gastric aspirates, although its biological role remains unknown [14,15]. Pivotally, in 2019, Zhang [2] et al. used mass spectrometry to detect the lactylation of histones in macrophages, revealing its direct role in regulating inflammatory responses. Subsequent investigations established that lactylation occurs widely across various tissues and cell types. It is particularly prominent in metabolically active muscle tissues, where it exhibits dynamic, specific, functional, and pathogenic characteristics [7,8,9,10,11,12,13].

## 3. Chemical Basis and Mechanisms of Lactylation

### 3.1. Chemical Basis of Lactylation

Protein lysine lactylation is a post-translational modification involving a lactate molecule that forms an amide bond with the ε-amino group of a lysine residue, resulting in Nε-lactyllysine. This modification increases the mass of the residue by 72 Da and reduces its net charge by +1 [2]. Compared to acetyl, the lactyl group is bulkier and contains a hydroxyl group, which may introduce a more pronounced steric hindrance [16]. Under high glycolytic flux (e.g., in muscle ischemia or during intense exercise), glucose is metabolized in the cytosol via glycolysis to generate pyruvate, which is subsequently reduced to lactate by lactate dehydrogenase A (LDHA), thereby establishing a favorable environment for protein lactylation [17]. L-lactate-mediated lactylation represents an epigenetic regulatory mechanism by which cells respond to glycolysis. It mainly involves the covalent attachment of a polar hydroxyl-bearing l-lactoyl group to lysine residues [18,19]. This modification not only significantly enhances the polarity and solubility of lysine residues but also influences protein function by modulating the protein’s spatial conformation, receptor binding ability, stability, and interactions with other molecules [20]. Moreover, innate immune activation and the accumulation of intestinal bacteria can be regulated through D-lactate mediated lactylation [21,22].

### 3.2. Lactylation Mechanisms

L-lactate-mediated lactylation is an enzymatic post-translational modification governed by three core mechanisms: “writing”, “erasing”, and “reading”. During the “writing” phase, L-lactate is activated to lactoyl-CoA by lactoyl-CoA synthetases (e.g., acetyl-CoA synthetase 2 (ACSS2), succinyl-CoA synthetase (GTPSCS)) [23,24]. The lactyl group is then transferred to specific lysine residues on histone or non-histone proteins by lactyltransferases, including lysine acetyltransferases lysine acetyltransferase 2 (KAT2), E1A-binding protein p300 (p300), CREB-binding protein (CBP), general control non-depressible 5 (GCN5), and Tat-interactive protein 60 (TIP60) [20]. Additionally, an alternative ATP-dependent mechanism has been reported, wherein aminoacyl-tRNA synthetase 1 (AARS1) and AARS2 catalyze the formation of a lactyl-AMP intermediate, which subsequently modifies specific target proteins to alter their function [8,25]. The “erasing” mechanism is primarily mediated by Zn^2+^-dependent histone deacetylases 1–3 (HDAC1–3) and NAD^+^-dependent deacetylases Sirtuin1-3 (SIRT1-3), which remove lactyl modifications via hydrolysis [26]. The regulatory mechanisms governing the activity of key enzymes involved in lactylation are summarized in Table 1. The “reading” mechanism involves specific recognition of lactylated proteins by dedicated readers. The known readers include brahma-related gene 1 (Brg1), which recognizes H3K18 lactylation via its bromodomain to regulate stem cell reprogramming [27]; tripartite motif-containing protein 33 (TRIM33), which binds H3K18 lactylation through its PHD-bromodomain to modulate transcription in late-stage M1 macrophage activation [28]; and double PHD fingers 2 (PHD2), which interacts with H3K14 lactylation via its tandem PHD fingers to activate oncogenes [29]. However, the physiological and pathological mechanisms of lactylation in muscle tissues and other systems remain to be fully elucidated.

**Figure 1:**
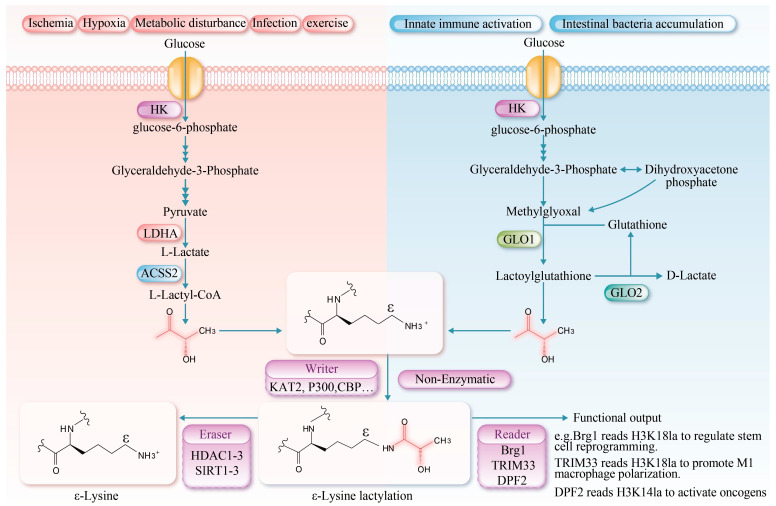
Protein lysine lactylation serves as a crucial bridge linking cellular metabolism and epigenetic regulation. Chemically, it involves the covalent binding of lactate to lysine residues via an amide bond, forming Nε-lactyllysine, which alters the protein’s charge, polarity, and spatial conformation, thereby influencing its function. The modification mechanisms are divided into two types: enzymatic and non-enzymatic. L-lactate-mediated lactylation is dynamically regulated by “writer” enzymes (e.g., acetyltransferases KAT2, p300 and CBP), “eraser” enzymes (e.g., deacetylases HDAC1-3 and SIRT1-3), and “reader” proteins. In contrast, D-lactate-mediated lactylation occurs non-enzymatically via lactoylglutathione. This modification is significantly enhanced under high glycolytic conditions and plays a broad role in regulating gene expression and cellular functions. La: lactylation, HK (Hexokinase); ACSS2 (Acetyl-CoA synthetase 2).

Recent studies have identified a non-enzymatic reaction in which an L-lactyl group is transferred from L-lactyl-CoA to lysine residues. This reaction is fundamentally chemical in nature, exhibiting clear time- and concentration-dependent characteristics. In vitro, it can modify a variety of proteins, including bovine serum albumin (BSA) in an enzyme-free system. Although this pathway is less efficient and less site-specific than its enzymatic counterparts, it represents a fundamental chemical mechanism that may regulate protein function in environments of locally high metabolite concentration. Furthermore, D-lactate-mediated lysine lactylation has been recognized as a significant, non-enzyme-dependent form of post-translational protein modification [38]. Furthermore, D-lactate-mediated lactylation is a significant non-enzymatic post-translational modification pathway. This process is initiated by methylglyoxal, a glycolytic intermediate primarily derived from the non-enzymatic degradation of phosphodihydroxyacetone and glyceraldehyde-3-phosphate [39]. During the catalysis of glyoxalase 1 (GLO1), methylglyoxal conjugates with glutathione to form lactoylglutathione. Lactoylglutathione serves as a lactyl donor, transferring the lactyl group to lysine residues on target proteins via a non-enzymatic mechanism, thereby facilitating lactylation. Subsequently, glyoxalase 2 (GLO2) catalyzes the hydrolysis of lactoylglutathione, regenerating glutathione for future catalytic cycles and releasing D-lactate [40]. Collectively, lactylation modifications constitute a crucial link between cellular metabolic states and epigenetic regulation (Figure 1).

## 4. Characteristics of Lactylation in Muscle Tissues

The muscles are a primary site for lactate production, particularly during high-intensity exercise or under hypoxic conditions, when anaerobic glycolysis is markedly activated, resulting in substantial lactate accumulation. This unique metabolic milieu makes muscle tissue an ideal model for studying lactylation. A locally high lactate concentration provides ample substrate for lysine lactylation, thereby driving a series of highly tissue-specific and dynamic modification processes. These modifications profoundly influence muscular energy homeostasis, cell fate, and disease progression, highlighting the direct role of the metabolic microenvironment in epigenetic regulation.

### 4.1. Dynamic Regulation of Lactylation in Muscle Tissue

The level of lactylation in muscle tissues is closely linked to lactate metabolism. Under resting conditions, skeletal muscle lactate concentration typically remains at 1 mM [41]. Conversely, during intense exercise, rapid glycolysis induces transient lactate accumulation, increasing concentrations to 5–10 mM [42]. This sharp increase promptly induces lactylation of both histone (e.g., H3K18) and non-histone proteins (e.g., pyruvate kinase M2) [43]. Notably, although blood lactate levels return to the baseline approximately 1 h after exercise cessation, protein lactylation in skeletal muscles (such as the soleus) peaks at 24 h post-exercise before gradually declining. After intense exercise, lactylation levels remain significantly elevated compared with resting controls, even at 48 and 72 h after exercise [7]. Zhang [2] et al. aptly termed this delayed response between lactylation dynamics and rapid blood lactate fluctuations as the “lactate clock”.

A similar time-delay phenomenon has been observed in postmortem muscles. Lactylation levels increased rapidly within the first two hours after death, which coincided with glycogen breakdown and lactate accumulation [44]. Although the lactate concentration and glycolytic potential peaked at four hours postmortem and subsequently declined, lactylation continued to increase, peaking at 8 h before decreasing significantly and stabilizing after 24 h, this pattern suggests delayed integration of metabolic signals [44]. This effect is likely linked to ATP availability, analogous to the mechanism of lactylation observed in tumor cells. The process is ATP-dependent at two key steps: first, ACSS2 consumes ATP to activate lactate into lactyl-CoA [23], and second, the lactyltransferase AARS1 utilizes ATP to form a lactyl-AMP intermediate, which subsequently transfers the lactyl group to specific target proteins [25]. As postmortem ATP depletes over time, reduced lactate dehydrogenase activity and insufficient lactyl-CoA availability lead to decreased lactylation levels; this effect is particularly evident in lactate-treated groups, indicating that ATP availability is crucial for maintaining the dynamic balance of protein lactylation [45].

### 4.2. Specificity of Lactylation in Muscle Tissue

Skeletal muscles consist of slow-twitch (type I) and fast-twitch (type II) fibers that exhibit distinct lactate metabolism and lactylation patterns due to their different metabolic characteristics and regulatory mechanisms. Type I fibers are rich in mitochondria and highly express aerobic metabolic enzymes that primarily rely on the oxidative metabolism of fat and glucose for energy, resulting in minimal lactate production [46]. However, they also express monocarboxylate transporter 1, which efficiently imports extracellular lactate for use as a substrate for oxidative phosphorylation [47]. This “consumer” characteristic enables type I fibers to display elevated lactylation levels following exercise. Research has demonstrated that after 30 min of running, although both fiber types exhibit lactate accumulation, type I fibers exhibit more pronounced lactylation at specific sites, including K336 of the pyruvate dehydrogenase E1 alpha 1 subunit (PDHA1) and K457/K458 of carnitine palmitoyltransferase 2 (CPT2) [8]. This pattern suggests that lactylation plays a crucial regulatory role in oxidative metabolism and endurance adaptation in type I muscle fibers.

In contrast, type II muscle fibers exhibit a characteristic glycolytic metabolic profile, characterized by high glycolytic enzyme activity, fewer mitochondria, and a primary dependence on anaerobic glycolysis for energy production [48]. These fibers can generate substantial lactate and rapidly export it via monocarboxylate transporter 4 (MCT4) [47]. As a result, type II fibers serve primarily as lactate “producers” and demonstrate relatively low levels of protein lactylation. Huang [7] et al. found that lactate levels were significantly elevated in the mouse soleus muscle, which is predominantly composed of oxidative fibers, at 24 h after an acute high-intensity interval training session. No comparable increase was detected in the gastrocnemius muscle, which is enriched with glycolytic fibers. Notably, other studies have indicated that neither acute nor chronic resistance or endurance training alter global lactylation levels in the human vastus lateralis muscle [49]. The potential influence of factors occurring outside the measured time points on skeletal muscle protein lactylation cannot be fully ruled out. Future human studies incorporating more frequent sampling are warranted to validate these findings. However, due to methodological limitations, it is challenging to assess the differences in fiber type-specific lactylation (type I vs. type II) in human muscle samples. Further investigations using animal models are required to address this. Information regarding key lactylation targets in muscle tissue can be found in Table 2.

### 4.3. Functional Diversity of Lactylation in Muscle Tissue

Lactylation plays a pivotal role in multifaceted and intricate regulatory networks governing energy metabolism in muscle cells. Its influence encompasses three major domains—glycolysis, lipid metabolism, and mitochondrial function—and it facilitates complex regulatory mechanisms under various physiological and pathological conditions.

#### 4.3.1. Lactylation Regulates Glucose and Lipid Energy Metabolism in Muscle Cells

Lactylation provides multilayered, precise control of energy metabolism in muscle cells and regulates glucose metabolism, lipid metabolism, and mitochondrial function through context-dependent mechanisms.

For glucose metabolism, lactylation enhances the glycolytic pathway by modifying key glycolytic enzymes and inducing epigenetic changes. Mechanistically, lactylation at site 678 of phosphofructokinase-M (PFKM) markedly increases its enzymatic activity [80]. Similarly, the K62 site of pyruvate kinase M2 (PKM2) promotes the formation of highly active tetramers that work in concert to amplify glycolytic flux [81]. In muscle cells, lactylation targets both rate-limiting glycolytic enzymes (PFKM and PKM2) and non-rate-limiting enzymes (fructose-bisphosphate aldolase A and phosphoglycerate kinase 1) [9]. The lactylation levels of these enzymes are positively correlated with their protein expression levels, suggesting that lactylation may enhance the stability or activity of glycolytic enzymes [64]. Epigenetically, Research has confirmed that histone H4K12 lactylation can be enriched in the promoter region of the key glycolytic factor PKM2. This modification enhances glycolytic activity and lactate production in microglial cells, thereby establishing a positive feedback loop [82].

The regulation of lipid metabolism by lactylation demonstrates distinct dual-phase characteristics with opposing effects under physiological and pathological conditions. Under physiological conditions, an 8-week high-intensity interval training study revealed a novel metabolic pathway of lipid catabolism by lactylation. The study demonstrated that exercise-induced lactate accumulation triggers lactylation of both fatty acid synthase (FASN) and ATP-citrate lyase (ACLY) in adipose tissue. Fatty acid synthase lactylation directly inhibits its enzymatic activity, thereby curtailing the biosynthesis of its downstream products, including palmitate and triglycerides [83]. In contrast, under pathological conditions such as hypertrophic obstructive cardiomyopathy, lactylation induces metabolic disturbances. Lactylation of short-chain acyl-CoA dehydrogenase (ACADS) and acetyl-CoA acetyltransferase 2 (ACAA2) inhibits their activity, directly impairing myocardial fatty acid β-oxidation [65]. Furthermore, studies in septic heart tissue and lipopolysaccharide-induced cultured cells have shown that the deacetylases Sirtuin 1 and Sirtuin 3 regulate the lactylation of rifunctional enzyme subunit alph (HADHA) protein at lysine 166 and 728. This lactylation modification inhibits HADHA’s enzymatic activity, thereby impairing mitochondrial fatty acid β-oxidation [66].

**Figure 2:**
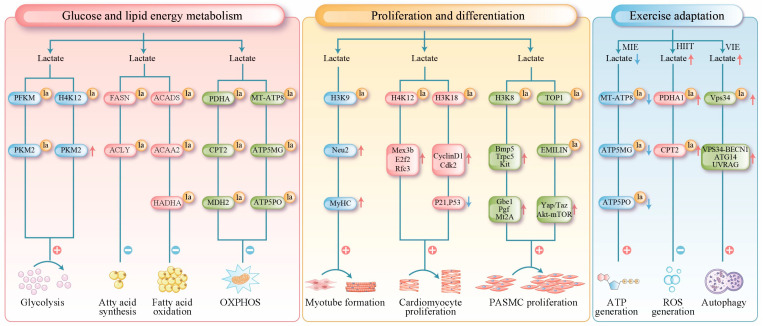
Lactylation in muscle tissue has been found to serve three primary functions. Red arrows indicate upregulation, and blue arrows indicate downregulation. Metabolic Regulation: It regulates glucose and lipid metabolism through direct modification of key metabolic enzymes (e.g., PFKM, FASN, SCAD). Furthermore, it suppresses oxidative phosphorylation by targeting mitochondrial proteins (e.g., PDHA1, CPT2, ATP synthase), thereby controlling processes from substrate utilization to terminal energy production. Epigenetic Regulation of Proliferation and Differentiation: Histone lactylation (e.g., H3K18la) modulates chromatin accessibility, directly activating genes involved in the cell cycle and muscle development to precisely coordinate the differentiation and proliferation of skeletal, cardiac, and smooth muscle cells. Exercise-Induced Protection: It limits excessive oxidation and prevents oxidative damage by inhibiting the activity of mitochondrial proteins such as PDHA1. Additionally, lactylation of VPS34 promotes autophagy, facilitating the clearance of damaged components and supporting cellular homeostasis. la (lactylation); MT-ATP8 (mitochondrially encoded ATP synthase membrane subunit 8); ATP5MG (ATP synthase membrane subunit g); ATP5PO (ATP synthase peripheral stalk subunit oscp); MIE (moderate-intensity exercise); HIIT (high-intensity exercise); VIE (vigorous-intensity exercise).

Lactate modulates mitochondrial metabolism by regulating mitochondrial protein lactylation. At the metabolic entry point, lactate produced under hypoxic conditions is specifically recognized and catalyzed by the lactyltransferase AARS2 within the mitochondrial matrix. AARS2 mediates the lactylation of endogenous PDHA at lysine 336 and exogenously expresses CPT2 at lysines 457/458, this modification inhibits the activity of both enzymes, thereby limiting the substrate availability for oxidative phosphorylation (OXPHOS). The deacetylase SIRT3 can reverse this lactylation to restore OXPHOS activity [8]. Some phenolic acid compounds (e.g., chlorogenic acid and gallic acid) may restore OXPHOS substrate supply by reducing the lactylation levels of these enzymes [84,85]. Within the oxidative pathway, the mitochondrial enzyme malate dehydrogenase 2 (MDH2) catalyzes the reversible conversion of malate to oxaloacetate. Dysfunction of MDH2 disrupts cellular energy supply by causing an accumulation of tricarboxylic acid cycle intermediates [86]. Lactylation of MDH2 at lysine 241 is thought to downregulate its activity, thereby limiting OXPHOS [67]. At the terminal energy production stage, lactylation of multiple mitochondrial ATP synthase subunits (e.g., MT-ATP8, ATP5MG, and ATP5PO) decreases their protein expression; this impairment ultimately compromises the efficiency of OXPHOS [50] (Figure 2).

#### 4.3.2. Lactylation Regulates Muscle Cell Proliferation and Differentiation

Lactylation serves as a key epigenetic mechanism in the regulation of muscle cell proliferation and differentiation.

In skeletal muscle, histone lactylation directly drives the differentiation of myoblasts. During differentiation, the promoter and enhancer regions of numerous genes associated with the terminal differentiation state acquire high levels of H3K18 lactylation. Treatment of myoblasts with lactate leads to upregulated expression of genes marked by specific H3K18 lactylation promoter peaks in myotubes. These genes are critically involved in skeletal muscle development and striated muscle cell differentiation [10]. These results suggest that lactate activates the expression of key genes by increasing histone lactylation in their promoter regions, thereby driving differentiation. Other investigations have shown that H3K9 lactylation accumulates in the enhancer region of *Neuraminidase 2* (*Neu2*). This enrichment competitively inhibits HDAC3, enhances chromatin accessibility, and activates *Neu2* transcription. Increased *Neu2* expression promotes myoblast differentiation into myotubes and enhances the expression of the differentiation marker myosin heavy chain (MyHC) [51]. Furthermore, the H3K18 lactylation level in macrophages has emerged as a potential biomarker for predicting muscle regenerative capacity [52].

In cardiac muscles, lactylation is closely associated with metabolic transitions during heart development [64]. High glycolytic activity in neonatal cardiomyocytes generates substantial amounts of lactate, driving the lactylation of histones, including H3K18 and H4K12. These modifications promote chromatin accessibility and activate cell cycle and DNA replication genes (e.g., *Mex3b*, *E2f2*, and *Rfc3*), thereby supporting cardiomyocyte proliferation [68]. Specifically, H4K12 lactylation downregulates *Vstm5* gene expression, thereby delaying the cell cycle progression. As the heart matures and metabolic patterns shift from glycolysis to fatty acid oxidation, lactylation levels decline along with the proliferative capacity [68]. In addition, studies show that exogenous 12-HEPE supplementation enhanced glycolysis via the Hippo-YAP pathway, subsequently promoting H3K18 lactylation; this modification downregulates the expression of cell cycle inhibition genes (e.g., *P21* and *P53*) and upregulates the expression of pro-proliferative genes, including *Cyclin D1 and Cdk2*, thereby reactivating the proliferative program in cardiomyocytes [69].

In smooth muscle, lactylation drives pathological vascular remodeling by promoting the abnormal proliferation of smooth muscle cells. In pulmonary arterial hypertension, hypoxia enhances glycolysis through the HIF-1α/PDK signaling axis, leading to increased lactate production. The accumulated lactate induces H3K18 lactylation. This modification enriches the promoter regions of pro-proliferative genes, including *Bmp5*, *Trpc*5, and *Gbe*1, thereby significantly upregulating their transcription and directly driving the proliferation of pulmonary arterial smooth muscle cells (PASMCs) [54]. H3K18 lactylation is also highly enriched in the promoter regions of genes, such as *Gbe1*, *Pgf*, and *Mt2A*, which upregulates their expression and promotes the proliferation of PASMCs [55]. Widespread non-histone lactylation also contributes to this process, for example, hyper-lactylation induces over-accumulation of topoisomerase 1 (TOP1) and deficiency of elastin microfibril interface-located protein 1 (EMILIN-1) in PASMCs, leading to the upregulation of Yap/Taz, Akt-mTOR, TGFβ1, and increased proliferation [56] (Figure 2).

#### 4.3.3. Lactylation Contributes the Adaptation of Skeletal Muscle to Exercise

Lactylation plays a key regulatory role in muscle adaptation to exercise by modulating multiple metabolic pathways and cellular functions.

Moderate-intensity exercise reduces lactate concentrations, thereby decreasing the lactylation of mitochondrial ATP synthase subunits (e.g., MT-ATP8, ATP5MG, and ATP5PO), enhancing oxidative phosphorylation efficiency, and ensuring adequate energy supply [50]. In contrast, during high-intensity exercise, lactate-mediated lactylation of mitochondrial PDHA and CPT2 inhibits their activity, thereby blocking acetyl-CoA production. This restriction of excessive oxidative phosphorylation prevents proton gradient collapse and excessive reactive oxygen species production, thereby protecting muscle cells from oxidative damage [8]. Furthermore, to mitigate post-vigorous-intensity exercise protein denaturation and mitochondrial damage, lactylation of the vacuolar protein sorting 34 (VPS34)-K356/K781 in skeletal muscle promotes autophagy and endolysosomal degradation, clearing damaged organelles and proteins to maintain cellular homeostasis [53].

Although lactate levels increase after exercise, some studies have reported no significant increase in protein lactylation in skeletal muscle after resistance training. For instance, despite a 3.5% increase in human vastus lateralis growth after 6 weeks of resistance training and 43.7% growth in mouse plantaris muscle following a 10-day overload-induced hypertrophy, these muscles showed no significant increase in nuclear or cytoplasmic protein lactylation but elevated blood and muscle lactate concentrations [49]. Potential explanations for this include: (1) exercise-induced lactate concentrations remain within physiological ranges, which are substantially lower than the levels required to induce lactylation in vitro; (2) limited availability of lactyl-CoA in muscle cells restricts its utility as a modification substrate; and (3) direct competition between lactylation and acetylation of lysine residues, as both modifications share regulatory enzymes (e.g., p300/CBP and HDAC1-3) and resistance training enhances acetylation, which may interfere with lactylation [20,49,87].

Collectively, these studies demonstrate that lactate functions not merely as a metabolic byproduct, but as a dynamic and precise signaling molecule. During exercise, it modulates muscle cells in real-time through lactylation. Under high-intensity conditions, lactate acts as a brake by inhibiting key enzymes to prevent energy system overload and oxidative damage, thereby protecting muscle tissue. At moderate intensity, it fine-tunes cellular metabolism by reducing its inhibitory effects on mitochondrial function, thereby enhancing energy production efficiency. Following strenuous exercise, lactate facilitates recovery by promoting autophagic clearance of damaged proteins and organelles. Interestingly, despite repeated elevations in lactate during long-term resistance training, widespread and persistent lactylation of muscle proteins has not been detected. This observation underscores the subtle nature of its regulation: rather than leaving permanent marks, lactylation likely constitutes a transient and precise mechanism that may cooperate or compete with other post-translational modifications, such as acetylation. Thus, the role of lactate in exercise adaptation is evolving from the outdated view of a “fatigue toxin” to a sophisticated signaling molecule central to the body’s intelligent adaptation to physical stress. Future research is needed to further elucidate how this small molecule orchestrates the processes that make muscle tissue stronger and more fatigue-resistant under diverse physiological conditions (Figure 2).

### 4.4. Central Mechanisms of Lactylation in Muscle Tissue Under Pathological Conditions

Lactylation not only regulates physiological processes such as muscle energy metabolism, exercise adaptation, and cell fate but is also closely linked to pathological conditions, including muscle aging, metabolic myopathies, and cardiovascular diseases.

#### 4.4.1. Lactylation in Muscle Aging

Lactylation also plays a crucial regulatory role in muscle aging. Studies have demonstrated that histone lactylation levels (e.g., H3K9, H3K14, and H3K18) are significantly reduced in aged skeletal muscles (such as the gastrocnemius muscle), which is closely associated with diminished glycolytic activity, decreased lactate production, and reduced NAD^+^ levels. Mechanistically, this decline in lactylation induces epigenetic reprogramming, directly lowering lactylation levels at the promoter regions of genes involved in DNA repair (e.g., *Pds5b*, *Park7*, and *Eya1*) and protein homeostasis (e.g., *Nedd4*, *Ubc*, and *Wwp1*), thereby suppressing their expression; this dysregulation accelerates muscle aging [11]. Studies have demonstrated that both exogenous lactate treatment, which increases lactylation levels, and supplementation with the NAD precursor nicotinamide, a broad-spectrum competitive inhibitor of NAD^+^-dependent Sirtuins, can effectively delay muscle aging. The underlying mechanism involves the modulation of histone acetylation and succinylation, as well as the inhibition of delactylation [11,88,89].

**Figure 3:**
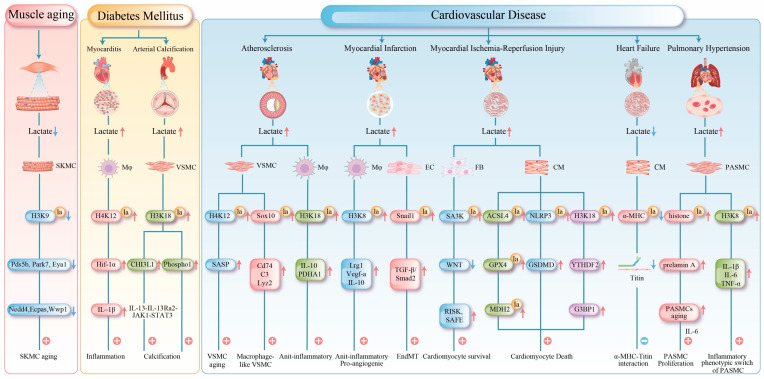
This figure summarizes the central mechanisms of protein lactylation in muscle tissue under pathological conditions. Red arrows indicate upregulation, and blue arrows indicate downregulation. 1. Muscle Aging: Reduced lactate production in aged muscle leads to decreased histone lactylation, which subsequently suppresses the expression of genes critical for DNA repair and protein homeostasis, thereby accelerating functional decline. 2. Metabolic Myopathies: Histone lactylation upregulates the expression of *HIF-1α*, *CHI3L1*, and *Phospho1*, exacerbating myocardial inflammation and promoting arterial calcification. 3. Cardiovascular Diseases: Atherosclerosis: Lactylation both accelerates vascular smooth muscle cell senescence by activating the senescence-associated secretory phenotype and promotes macrophage polarization to exert protective effects. Myocardial Infarction: Early-phase lactylation promotes the expression of repair genes, while late-phase lactylation induces EndMT, exacerbating fibrosis. Myocardial Ischemia-Reperfusion Injury: Lactylation exhibits dual regulatory roles through different targets, simultaneously activating protective pathways while promoting ferroptosis and inflammatory responses. Heart Failure: Impaired lactylation of α-MHC compromises myocardial contractility. Pulmonary Hypertension: Lactylation drives PASMCs and contributes to pulmonary vascular remodeling by modulating cellular senescence and inflammatory phenotypes. SKMC (Skeletal Muscle Cell); Mφ (Macrophage); EC (Endothelial Cell); FB (Fibroblast); CM (cardiac muscle cell); IL-1β (Interleukin-1β); Cd74 (cluster of differentiation 74); C3 (complement component 3); Lyz2 (lysozyme 2); TGF-β (transforming growth factor beta); Smad2 (smad family member 1); GSDMD (Gasdermin D); G3BP1 (Ras GTPase-activating protein-binding protein 1).

Exercise is an effective strategy for counteracting muscle aging. Compared with sedentary controls, middle-aged mice subjected to a two-month treadmill running regimen (9 m/min, 30 min per session) significantly enhances glycolytic activity in skeletal muscles and increases histone lactylation. This exercise-induced “lactylation reprogramming” reactivates DNA repair and proteostasis genes, alleviating muscle dysfunction. Concurrently, running remodels muscle cellular composition by markedly increasing the proportion of IIb-type myonuclei and muscle stem cells while reducing adipocyte accumulation. This optimized cellular architecture restructures intercellular communication networks and coordinately activates pathways related to glycolysis, oxidative phosphorylation, and myofiber regeneration. Ultimately, these adaptations comprehensively improve muscle metabolic capacity, contractile function, and regenerative potential, thereby effectively counteracting the effects of aging [11,90] (Figure 3).

#### 4.4.2. Lactylation in Metabolic Myopathies

Lactylation exhibits distinct physiological and pathological roles in metabolic regulation, which explains its apparent functional paradox. During acute stimuli, such as exercise, a transient surge in lactate levels enhances insulin sensitivity via the AMPK/PGC-1α/GLUT4 signaling axis; this is accompanied by rapid, short-term protein lactylation, which further promotes insulin sensitivity by augmenting glycolytic flux [43]. In contrast, chronic diseases such as obesity and diabetes induce persistent hyperlactatemia, leading to sustained protein lactylation. This chronic modification suppresses mitochondrial biogenesis genes, resulting in mitochondrial dysfunction and exacerbating insulin resistance [12,43,57]. Therefore, it is the abnormal and sustained elevation of lactate—rather than its transient physiological fluctuations—that serves as a common pathogenic driver of dysfunction in skeletal, cardiac, and vascular smooth muscle.

In skeletal muscles, lactylation is closely associated with insulin resistance and type 2 diabetes. A study involving 15 lean and 14 obese adults revealed significantly higher protein lactylation levels in the skeletal muscles of obese individuals, which positively correlated with the degree of insulin resistance [12]. Under insulin-resistant conditions, intracellular glucose metabolism is dysregulated, preferentially shifting toward lactate production [91]. This may persistently elevate lactylation levels and disrupt glucose metabolic homeostasis in skeletal muscle. Furthermore, the observed decrease in lactylation (e.g., H3K18 lactylation) following metformin treatment, which improves insulin resistance, corroborates its crucial role in this process [92].

The pathogenic effects of lactylation occur in other muscle tissues. In diabetic cardiomyopathy, elevated levels of free fatty acids stimulate cardiomyocytes to produce excess lactate, which is exported via MCT4 into the microenvironment. This promotes H4K12 lactylation in cardiac macrophages. MCT4 inhibition reverses cardiac macrophage H4K12 lactylation and alleviates cardiac inflammation [70]. Lactylation directly promotes pathological vascular calcification in diabetes. Under high-glucose conditions, global histone lactylation levels (e.g., H3K18 and H3K14) increase in muscle cells. Specifically, H3K18 lactylation upregulates *chitinase-3-like protein 1* (*CHI3L1*) expression, activating the IL-13Rα2/JAK1/STAT3 signaling pathway and driving the transition of vascular smooth muscle cells (VSMCs) to an osteogenic phenotype [57]. Furthermore, H3K18 lactylation enrichment in the *phosphatase 1* (*Phospho1*) promoter upregulates its expression and accelerates arterial calcification [58] (Figure 3).

#### 4.4.3. Lactylation in Cardiovascular Diseases

(1) Atherosclerosis: The progression of atherosclerosis is closely linked to metabolic dysregulation and phenotypic changes in VSMCs and macrophages [93]. In VSMCs, cellular senescence is a key driver of atherosclerosis [94]. Senescent VSMCs undergo a metabolic shift from oxidative phosphorylation to aerobic glycolysis. The mitochondrial protein tumor necrosis factor receptor-associated protein 1 (TRAP1) is overexpressed in senescent VSMCs, which promotes glycolysis and suppresses the tricarboxylic acid cycle. Conversely, TRAP1 knockout reduces lactate levels and histone H4 lactylation. Further investigations have revealed that TRAP1 promotes H4K12 lactylation by downregulating HDAC3 via lactate, thereby activating senescence-associated secretory phenotype (SASP) transcription and accelerating VSMC senescence and atherosclerosis progression [59]. In advanced atherosclerotic plaques, chronic inflammation induces the transdifferentiation of some VSMCs into macrophage-like cells, a process associated with sex-determining region Y (SRY)-related HMG-box gene 10 (Sox10) lactylation, which exacerbates intimal inflammation and promotes vulnerable plaque formation [60].

However, lactylation can promote anti-inflammatory effects in macrophages. H3K18 lactylation activates the transcription of the reparative genes *Interleukin-10* (*IL-10)* and pyruvate metabolic gene *pyruvate dehydrogenase alpha* (*PDHA)*, promoting the transition from pro-inflammatory M1 to anti-inflammatory M2 macrophages, thereby attenuating atherosclerosis progression [61]. MCT4 plays a critical role in this process; its deficiency leads to intracellular lactate accumulation, which enhances H3K18 lactylation and facilitates macrophage polarization [61].

(2) Myocardial Infarction: Myocardial infarction (MI), as a severe clinical manifestation of atherosclerosis, is characterized by coronary artery occlusion that drastically reduces myocardial blood supply and impairs peripheral organ perfusion. This condition frequently induces hyperlactatemia, a metabolic disorder closely associated with progressive myocardial damage [95]. During the early phase of MI (day 1), lactate promotes H3K18 lactylation in monocytes and macrophages, thereby enhancing the transcription of repair-related genes (e.g., *Lrg1*, *Vegf-α*, and *IL-10*). Through their anti-inflammatory and pro-angiogenic activities, these genes foster a microenvironment conducive to tissue repair. Elevated H3K18 lactylation suppresses detrimental inflammation and improves cardiac function post-MI [71]. However, lactate is not entirely beneficial. In the later stage of MI (day 6), it may also promote Snail family transcriptional repressor 1 (Snail1) lactylation, activating the TGF-β/Smad2 signaling pathway and driving endothelial–mesenchymal transition (EndMT), which ultimately exacerbates myocardial fibrosis [32].
(3) Myocardial Ischemia-Reperfusion Injury: Myocardial ischemia-reperfusion injury (MIRI) is a secondary injury that occurs after blood flow restoration after post-myocardial infarction [96]. During MIRI, the cardiac energy metabolism shifts toward glycolysis, resulting in ATP depletion and lactate accumulation [97]. This subsequently induces protein lactylation, which exerts complex effects on cardiomyocyte survival. On the one hand, lactylation exhibits protective roles; heat shock protein A12A supports cardiomyocyte survival under hypoxia/reoxygenation by maintaining histone H3 lactylation and aerobic glycolytic homeostasis, and its deficiency exacerbates cardiac dysfunction [72]. Fibroblast-derived lactylated Serpina3k (SA3K) inhibits the WNT signaling pathway and activates the reperfusion injury salvage kinase (RISK) and survivor activating factor enhancement (SAFE) signaling pathways, reducing cardiomyocyte apoptosis [73].

In contrast, lactylation promotes injury progression. During MIRI, lactate induces the lactylation of the key ferroptosis regulators acyl-CoA synthetase long chain family member 4 (ACSL4)-K83 and glutathione peroxidase 4 (GPX4)-K21/K228, thereby enhancing and reducing their stability, respectively, which collectively exacerbate lipid peroxidation and ferroptosis [74,75]. MDH2-K24 lactylation impairs mitochondrial function, creating a favorable environment for cardiomyocyte ferroptosis [67]. Additionally, lactate promotes NLR family pyrin domain containing 3 (NLRP3)-K245 lactylation, facilitating inflammasome assembly and activation to induce cardiomyocyte death [76]. Furthermore, lactate can promote cardiomyocyte death by improving H3K18 lactylation levels and elevating intracellular N6-methyladenosine RNA-binding protein YTHDF2 [77].

In summary, lactylation plays a dual role in MIRI; under specific conditions, it confers cardioprotection by maintaining glycolytic homeostasis and activating protective signaling pathways, whereas in other contexts, it exacerbates injury by promoting ferroptosis and inflammatory responses. These contradictory roles likely stem from the differential modulation of distinct target proteins, coupled with their cell type-specific functions and molecular context, highlighting the complexity and context-dependent nature of MIRI pathogenesis.

(4) Heart Failure: Lactylation exerts dual regulatory effects in heart failure development, with its impact being highly target- and stage-specific. During heart failure progression, metabolic reprogramming increases lactate efflux and reduces intracellular lactate levels, consequently diminishing the lactylation of key proteins [98]. In both murine models and human patients, decreased lactylation at K1897 of α-myosin heavy chain (α-MHC) impairs its interaction with titin, compromising sarcomeric integrity and reducing contractility. Moreover, this modification deficiency upregulates the expression of the fibrotic markers alpha-smooth muscle actin and type I collagen, further deteriorating cardiac function. Experimentally, restoring α-MHC-K1897 lactylation by elevating lactate concentration or inhibiting lactate efflux effectively improves cardiac performance [78].

However, lactylation results in complex cardiac effects. In a mouse model of cardiac hypertrophy, elevated H3K18 lactylation levels in cardiomyocytes promoted pathological hypertrophy, whereas decreased H3K18 lactylation levels significantly attenuated this condition [79]. This indicates that the biological effects of lactylation vary substantially depending on the specific modification site and pathological stage. Collectively, these findings suggest that precise modulation of site-specific lactylation may offer novel metabolic intervention strategies for heart failure treatment.

(5) Pulmonary Hypertension: In pulmonary hypertension, lactylation contributes to pulmonary vascular remodeling by directly promoting smooth muscle proliferation and regulating cellular senescence and inflammatory phenotypes [54,55,56]. Lactylation modulates the senescence of PASMCs in pulmonary hypertension models. The accumulation of the senescence-associated prelamin A and the subsequent increase in Interleukin-6 (IL-6) secretion collectively create a microenvironment that promotes PASMC proliferation, and histone lactylation is implicated in driving this process. Lactylation plays a critical role in regulating inflammation [62]. The long non-coding RNA UNC5B-AS1 remodels cellular metabolism by suppressing glycolysis and enhancing oxidative phosphorylation, thereby reducing lactate levels and H3K18 lactylation enrichment at the pro-inflammatory genes, such as *IL-1β*, *IL-6*, and *TNF-α*. This mechanism attenuates the transition of PASMCs to a pro-inflammatory phenotype and inhibits the formation of a pro-inflammatory vascular microenvironment [63].

These findings expand our understanding of lactylation in PH, revealing its novel regulatory roles in cellular senescence and inflammation as well as highlighting potential therapeutic targets for PH treatment strategies focused on metabolic-epigenetic crosstalk (Figure 3).

## 5. Novel Strategies Targeting Lactylation for Muscular Disorders

Dysregulated lactylation is implicated in various pathological processes, including muscle aging, metabolic myopathies, and cardiovascular diseases, making the targeted modulation of lactylation a promising therapeutic strategy. The dynamic balance of lactylation in muscle tissue is maintained by specific “writer” and “eraser” enzymes: acetyltransferases (e.g., KAT2A, P300, and CBP) catalyze lactylation [20], while de-modifying enzymes (e.g., HDAC1-3 and SIRT1-3) remove these modifications [26].

To date, various small molecule compounds that selectively target these modifying enzymes have been identified. For instance, C646, A-485, and curcumin inhibit P300/CBP activity [78,99]; trichostatin A and suberoylanilide hydroxamic acid suppress HDAC1-3, and nicotinamide blocks the delactylation function of SIRT1-3 [13,71]. Therefore they serve as valuable molecular tools for modulating lactylation levels. Alternatively, targeting the upstream lactate production pathways has gained significant research attention. LDHA inhibitors (e.g., FX-11, GSK2837808A, and vitamin C) directly block lactate synthesis, whereas compounds such as stiripentol and gossypetin function as broad-spectrum dual LDH inhibitors [58,95]. Moreover, 2-deoxyglucose and lonidamine indirectly reduce lactate production by inhibiting hexokinase, whereas dichloroacetate reduces lactate levels by promoting pyruvate entry into the tricarboxylic acid cycle [12,100].

These interventions modulate lactylation through multiple mechanisms, thereby opening new therapeutic avenues for muscular disorders via metabolic regulation.

## 6. Conclusions and Future Perspectives

This review systematically examined the central role and regulatory mechanisms of lactylation in muscle physiology and pathology. As an emerging metabolic sensing mechanism, lactylation bridges glycolytic flux through gene transcription, protein function, and cell fate determination. Under physiological conditions, lactylation regulates the energy metabolism, proliferation, and adaptation of muscle cells, whereas dysregulated lactylation directly contributes to the pathogenesis of muscle aging, metabolic myopathies, and cardiovascular diseases.

Although significant progress has been achieved in this field, several challenges remain unresolved. First, lactylation exhibits dual—sometimes opposing—effects across different muscle types and disease stages, indicating high context-dependency. Future studies should employ single-cell sequencing and site-specific modification analyses to map the functional landscape of lactylation precisely. Second, the identification and functional understanding of lactylation “reader” proteins are still in their infancy and represent a key direction for mechanistic research. Finally, although existing targeting strategies (e.g., modifier enzyme inhibitors and lactate production inhibitors) have shown conceptual promise, their tissue specificity, temporal efficacy, and potential off-target effects require further evaluation using preclinical models.

## Figures and Tables

**Table 1:** Regulatory information on lactylation-modifying enzymes.

Enzymes	Regulatory Factors	Response to Physiological /Pathological States	References
Lactyl-CoA synthetase			
ACSS2	Signal initiation: The EGFR/ERK signaling pathway is activated, leading to the phosphorylation of ACSS2 at serine-267. Conformational change and translocation: PIN1 recognizes the phosphorylated site, induces a conformational change in ACSS2, and facilitates its nuclear translocation. Substrate requirements: The catalytic reaction requires the collective participation of lactate, CoA, and ATP.	High metabolic demand: In glycolytically active cells (tumor cells), they can respond to tumor microenvironmental changes such as growth factor signaling, hypoxia, and lactate accumulation.	[23]
GTPSCS	Nuclear localization signal (NLS): The NLS on the G1 subunit determines nuclear translocation. K73 acetylation on G2 subunit: This modification is a prerequisite for the interaction between GTPSCS and p300. Structural integrity: Substrate binding induces conformational rearrangement in the G2 subunit, affecting enzymatic activity. Substrate dependence: Lactate influences its catalytic activity.	High metabolic demand: In glycolysis-active tumor cells, lactate accumulation and increased levels of GTP/CoA lead to substantially enhanced activity.	[24]
Lysine lactyltransferase			
P300/CBP	Post-translational modification: Phosphorylation can enhance its activity. Protein-protein interaction: Binding to specific transcription factors can guide it to target sites. Substrate dependence: Lactate concentration influences its catalytic activity.	Metabolic reprogramming: When cellular metabolism shifts from oxidative phosphorylation to glycolysis, the enzyme may show a preferential tendency to catalyze lactylation.	[30,31,32]
AARS1/2	Substrate concentration: Elevated lactate levels enhance its lactyltransferase activity. Competitive inhibition: β-alanine competes with lactate for binding. ATP dependency: The reaction is ATP-dependent.	Exercise/hypoxia: The decrease in oxygen partial pressure in muscles leads to an increase in AARS2 protein levels. Tumorigenesis: AARS1 acts as a lactate sensor, converting metabolic signals into protein modification signals.	[8,25]
Lysine delactylase			
HDAC1-3	Constitutive activity: Its activity is regulated by subcellular localization, complex assembly, and phosphorylation. Zn^2+^-dependent catalytic activity: Maintained by Zn^2+^ and can be inhibited by chelators such as TSA (Trichostatin A). Direct inhibition by lactate: Evidence suggests that lactate itself may suppress its activity.	Constitutive regulation: As a fundamental delactylase, it likely maintains the baseline balance of lactylation modifications. Pathological State: In conditions such as atherosclerosis, its activity is altered.	[26,33,34]
SIRT1-3	NAD^+^/NADH ratio: The most critical regulatory factor. Elevated NAD^+^ activates the enzyme, while elevated NADH inhibits it. Direct inhibition by lactate: Evidence suggests that lactate itself may suppress its activity. Transcriptional and expression levels: Regulated by energy status (e.g., via AMPK) and other factors.	Energy sensing: During low-energy states (fasting or caloric restriction), the enzymatic activities of SIRT1-3 are enhanced. Pathological state: In heart failure, decreased NAD^+^ levels result in diminished delactylation capacity.	[35,36,37]

**Table 2:** Key lactylation site in muscle tissues.

Cell Type	Lactylation Site	Target Gene/ Pathway	Function	Reference
SKMC	PKM2, PKFM	/	Linked to glycolytic metabolism.	[9]
SKMC	PDHA1 K336, CPT2 K457/458	/	Inhibiting acetyl-CoA generation from both glucose and fatty acids.	[8]
SKMC	MT-ATP8, ATP5MG, ATP5PO	/	Suppressing the expression of related proteins to restrict oxidative phosphorylation.	[50]
SKMC	H3K9	*Neu2*	Driving myoblast differentiation into myotubes.	[51]
Macrophage	H3K18	/	Forecasting muscle regeneration potential based on injury biomarkers.	[52]
SKMC	VPS34 K356/K781	VPS34-BECNI, ATG14, UVRAG	Promoting autophagy and endo-lysosomal degradation.	[53]
SKMC	H3K9	*Pds5b*, *Park7b*, *Eya1*, *Nedd4*, *Ubc*, *Wwp1*	Activating DNA repair and proteostasis pathways to counteract muscle aging.	[11]
HSKMC	/	/	Linked to the development of insulin resistance.	[12]
PASMC	H3K18	*Bmp5*, *Trpc5*, *Gbe1*	Driving the proliferation of PASMCs and vascular remodeling.	[54]
PASMC	H3K18	*Gbe1*, *Pgf*, *Mt2A*, *Ythdf2*, *Gys1*	Driving the proliferation of PASMCs.	[55]
PASMC	TOP1, EMILIN-1	Yap/Taz, Akt-mTOR	Promoting PASMCs proliferation and survival.	[56]
VSMC	H3K18	*CHI3L1*	Inducing osteogenic transdifferentiation of VSMC.	[57]
VSMC	H3K18	*Phospho1*	Driving vascular calcification and regulating related gene expression.	[58]
VSMC	H4K12	SASP	Driving smooth muscle cell senescence and promoting disease development.	[59]
VSMC	Sox10	Cd74, C3, Lyz2	Inducing macrophage-like phenotypic switching in vascular smooth muscle cells	[60]
Macrophage	H3K18	*PDHA*, *IL-10*	Inducing M1 macrophage polarization.	[61]
PASMC	Histone	prelamin A	Elevating IL-6 secretion which drives proliferation of PASMC.	[62],
PASMC	H3K18	*IL-1β*, *IL-6*, *TNF-α*	Attenuating pro-inflammatory phenotypic switching in pulmonary artery smooth muscle cells.	[63]
Cardiomyocyte	PKM2, PKFM	/	Linked to glycolytic metabolism.	[64]
Cardiomyocyte	SCAD, ACAA2	/	Suppressing mitochondrial β-oxidation of fatty acids.	[65]
Cardiomyocyte	HADHA	/	Suppressing mitochondrial β-oxidation of fatty acids.	[66]
Cardiomyocyte	MDH2 K241	/	Participating in the regulation of mitochondrial function and cell death.	[67]
Cardiomyocyte	H4K12	*Mex3b*, *E2f2*, *Rfc3*	Promoting proliferative capacity of cardiomyocytes.	[68]
Cardiomyocyte	H3K18	*CyclinD1*, *Cdk2* *P21*, *P53*	Activating the proliferation program in cardiomyocytes.	[69]
Macrophage	H4K12	HIF-1α	Reduced infiltration of HIF-1α-positive and IL-1β-positive inflammatory macrophages in the heart.	[70]
Cardiomyocyte	H3K18	*Lrg1*, *Vegf-a*, *IL-10*	Regulating monocyte–macrophage function; participating in the early and remote activation of genes.	[71]
Endothelial cell	Snail1	TGF-β/Smad2	Activating TGF-β/Smad2pathway.	[32]
Cardiomyocyte	H3K56	/	Supports cardiomyocyte survival under hypoxia/reoxygenation	[72]
Fibroblast	SA3K K351	RISK/SAFE, WNT	Enhancing the protein stability of SA3K and reducing cardiomyocyte apoptosis.	[73]
Cardiomyocyte	ACSL4 K83	/	Inducing lipid peroxidation-driven ferroptosis in cardiomyocytes.	[74]
Cardiomyocyte	GPX4 K21/K228	/	Sensitizing cardiomyocytes to ferroptosis through GPX4 inhibition.	[75]
Cardiomyocyte	NLRP3 K245	GSDMD	Promoting cardiomyocyte pyroptosis and aggravating injury. Mediating injury through the LDHA-NLRP3 pathway.	[76]
Cardiomyocyte	H3K18	YTHDF2	Participating in exercise-induced physiological cardiac hypertrophy and I/R injury repair.	[77]
Cardiomyocyte	α-MHC K1897	Titin	Maintaining sarcomere structure and function.	[78]
Cardiomyocyte	H3K18	/	Driving pathological cardiac hypertrophy.	[79]

## Data Availability

No new data were created or analyzed in this study.

## Abbreviations

The following abbreviations are used in this manuscript:

HK	Hexokinase
LDHA	Lactate dehydrogenase A
ACSS2	Acetyl-CoA synthetase 2
GTPSCS	Succinyl-CoA synthetase
GLO1	Glyoxalase 1
GLO2	Glyoxalase 2
KAT2A	Lysine acetyltransferase 2A
P300	E1A binding protein p300
CBP	CREB-binding protein
GCN5	General control non-depressible 5
TIP60	Tat-interactive protein 60
AARS1/2	Aminoacyl-tRNA synthetase 1/2
HDAC1-3	Histone deacetylase 1-3
SIRT1-3	Sirtuin1-3
Brg1	Brahma-related gene 1
TRIM33	Tripartite motif-containing 33
DPF2	Double PHD fingers 2
MCT4	Monocarboxylate transporter 4
PFKM	Phosphofructokinase-M
PKM2	Pyruvate kinase M2
FASN	Fatty acid synthase
HIF-1α	Hypoxia-inducible factor-1α
ACLY	ATP-citrate lyase
ACADS	Short-chain acyl-CoA dehydrogenase
ACAA2	Acetyl-CoA acyltransferase 2
HADHA	Rifunctional enzyme subunit alph
PDHA1	pyruvate dehydrogenase E1 alpha 1 Subunit
CPT2	Carnitine palmitoyltransferase 2
MDH2	Malate dehydrogenase 2
MT-ATP8	Mitochondrially encoded ATP synthase membrane subunit 8
ATP5MG	ATP synthase membrane subunit g
ATP5PO	ATP synthase peripheral stalk subunit oscp
OXPHOS	Oxidative phosphorylation
*Neu2*	*Neuraminidase 2*
MyHC	Myosin heavy chain
*Mex3b*	*Mex-3bRNA binding family member b*
*E2f2*	*E2F transcription factor 2*
*Rfc3*	*Replication factor C subunit 3*
*Cdk2*	*Cyclin-dependent kinase 2*
*P21*	*Cyclin-dependent kinase 1A*
*P53*	*Tumor protein p53*
*Bmp5*	*Bone morphogenetic protein 5*
*Trpc5*	*Transient receptor potential cation channel subfamily C member 5*
*Kit*	*V-kit Hardy–Zuckerman 4 feline sarcoma viral oncogene homolog*
*Gbe1*	*1,4-α-Glucan branching enzyme*
*Pgf*	*Placental growth factor*
*Mt2A*	*Metallothionein 2A*
ACADS	Acyl-CoA dehydrogenase, short chain
TOP1	Topoisomerase I
EMILIN-1	Elastin microfibril interface protein 1
VPS34	Vacuolar protein sorting 34
BECN1	Beclin 1
AGT14	Autophagy related angiotensinogen14
UVRIG	UV radiation resistance associated gene
SKMC	Skeletal muscle cell
Mφ	Macrophage
VSMC	Vascular smooth muscle cell
*Pds5b*	*Pds5 cohesin associated factor b*
*Park7*	*Parkinsonism associated deglycase 7*
*Eya1*	*Eyes absent homolog 1*
*Nedd4*	*Neural precursor cell expressed development down-regulated 4*
*Wwp1*	*Ww domain containing E3 ubiquitin protein ligase*
*Ecpas*	*Erythrocyte coproporphyrinogen oxidase*
*CHI3L1*	*Chitinase-3-like protein 1*
*Phospho1*	*Phosphoethanolamine*
TRAP1	Tumor necrosis factor receptor-associated protein 1
SASP	Senescence-associated secretory phenotype
Sox10	Sex-determining region Y (SRY)-related HMG-box gene 10
*IL-10*	*Interleukin-10*
*PDHA*	*Pyruvate dehydrogenase alpha*
Cd74	Cluster of differentiation 74
C3	Complement component 3
Lyz2	lysozyme 2
MI	Myocardial infarction
*Lrg1*	*Leucine-rich alpha-2-glycoprotein 1*
*EC*	*Endothelial cell*
Snail1	Snail family transcriptional repressor 1
TGF-β	Transforming growth factor beta
Smad2	Smad family member 2
EndMT	Endothelial–mesenchymal transition
MIRI	Myocardial ischemia-reperfusion injury
FB	Fibroblast
SA3K	Serpina3k
WNT	Wingless-type MMTV integration site family, Member
RISK	Reperfusion injury salvage kinase
SAFE	Survivor activating factor enhancement
CM	Cardiac muscle cell
ACSL4	Acyl-CoA synthetase long chain family member 4
GPX4	Glutathione peroxidase 4
NLRP3	NOD-like receptor protein 3
GSDMD	Gasdermin D
YTHDF2	YTH domain-containing family protein 2
G3BP1	Ras GTPase-activating protein-binding protein 1
PASMC	Pulmonary arterial smooth muscle cell
α-MCH	α-Myosin heavy chain
*IL-1β*	*Interleukin-1β*
*IL-6*	*Interleukin-6*
*TNF-α*	*Tumor necrosis factor alpha*

## References

[1] Rabinowitz J.D., Enerbäck S. (2020). Lactate: The ugly duckling of energy metabolism. Nat. Metab..

[2] Zhang D., Tang Z., Huang H., Zhou G., Cui C., Weng Y., Liu W., Kim S., Lee S., Perez-Neut M. (2019). Metabolic regulation of gene expression by histone lactylation. Nature.

[3] Seheult J., Fitzpatrick G., Boran G. (2017). Lactic acidosis: An update. Clin. Chem. Lab. Med..

[4] Dartiguelongue J.B. (2024). Biological significance and clinical utility of lactate in sepsis. Arch. Argent. Pediatr..

[5] Dietrich D., Elzinga G. (1992). ATP formation and energy demand in anoxic heart muscle of the rabbit. Am. J. Physiol. Heart Circ. Physiol..

[6] Stephens N.L., Kroeger E.A., Loh W. (1977). Intracellular pH in hypoxic smooth muscle. Am. J. Physiol.-Endocrinol. Metab..

[7] Huang W., Su J., Chen X., Li Y., Xing Z., Guo L., Li S., Zhang J. (2023). High-intensity interval training induces protein lactylation in different tissues of mice with specificity and time dependence. Metabolites.

[8] Mao Y., Zhang J., Zhou Q., He X., Zheng Z., Wei Y., Zhou K., Lin Y., Yu H., Zhang H. (2024). Hypoxia induces mitochondrial protein lactylation to limit oxidative phosphorylation. Cell Res..

[9] Wu Z., Chai Z., Cai X., Wang J., Wang H., Yue B., Zhang M., Wang J., Wang H., Zhong J. (2024). Protein Lactylation Profiles Provide Insights into Molecular Mechanisms Underlying Metabolism in Yak. J. Agric. Food Chem..

[10] Galle E., Wong C.-W., Ghosh A., Desgeorges T., Melrose K., Hinte L.C., Castellano-Castillo D., Engl M., de Sousa J.A., Ruiz-Ojeda F.J. (2022). H3K18 lactylation marks tissue-specific active enhancers. Genome Biol..

[11] Meng F., He J., Zhang X., Lyu W., Wei R., Wang S., Du Z., Wang H., Bi J., Hua X. (2025). Histone Lactylation Antagonizes Senescence and Skeletal Muscle Aging by Modulating Aging-Related Pathways. Adv. Sci..

[12] Maschari D., Saxena G., Law T.D., Walsh E., Campbell M.C., Consitt L.A. (2022). Lactate-induced lactylation in skeletal muscle is associated with insulin resistance in humans. Front. Physiol..

[13] Xie M., Kong Y., Tan W., May H., Battiprolu P.K., Pedrozo Z., Wang Z.V., Morales C., Luo X., Cho G. (2014). Histone deacetylase inhibition blunts ischemia/reperfusion injury by inducing cardiomyocyte autophagy. Circulation.

[14] Angata T., Varki A. (2002). Chemical diversity in the sialic acids and related α-keto acids: An evolutionary perspective. Chem. Rev..

[15] Muthana S.M., Campbell C.T., Gildersleeve J.C. (2012). Modifications of glycans: Biological significance and therapeutic opportunities. ACS Chem. Biol..

[16] Fu Q., Cat A., Zheng Y.G. (2023). New histone lysine acylation biomarkers and their roles in epigenetic regulation. Curr. Protoc..

[17] Wang Z., Zhu L. (2025). New insights into lactate in exercise adaptations: Does protein lactylation play a role?. Am. J. Physiol.-Endocrinol. Metab..

[18] Zhang D., Gao J., Zhu Z., Mao Q., Xu Z., Singh P.K., Rimayi C.C., Moreno-Yruela C., Xu S., Li G. (2025). Lysine L-lactylation is the dominant lactylation isomer induced by glycolysis. Nat. Chem. Biol..

[19] Zhao L., Qi H., Lv H., Liu W., Zhang R., Yang A. (2025). Lactylation in health and disease: Physiological or pathological?. Theranostics.

[20] Wang Z.M., Yu Q.W., Wang C., Wang S.H., Wang P., Zhang L.R., Han S.N. (2025). Lactylation in Cardiovascular Diseases: Current Progress and Perspectives. J. Am. Heart Assoc..

[21] Zhao Q., Wang Q., Yao Q., Yang Z., Li W., Cheng X., Wen Y., Chen R., Xu J., Wang X. (2025). Nonenzymatic lysine D-lactylation induced by glyoxalase II substrate SLG dampens inflammatory immune responses. Cell Res..

[22] Zang Y., Zhang J., Xia M., Wang A., Fan Z., Han Y., Zhang H., Wang S., Niu Z., Wu J. (2024). D-lactate derived from intestinal bacteria drives lysine D-lactylation to modulate transcription in liver cells. bioRxiv.

[23] Zhu R., Ye X., Lu X., Xiao L., Yuan M., Zhao H., Guo D., Meng Y., Han H., Luo S. (2025). ACSS2 acts as a lactyl-CoA synthetase and couples KAT2A to function as a lactyltransferase for histone lactylation and tumor immune evasion. Cell Metab..

[24] Liu R., Ren X., Park Y.E., Feng H., Sheng X., Song X., AminiTabrizi R., Shah H., Li L., Zhang Y. (2025). Nuclear GTPSCS functions as a lactyl-CoA synthetase to promote histone lactylation and gliomagenesis. Cell Metab..

[25] Zong Z., Xie F., Wang S., Wu X., Zhang Z., Yang B., Zhou F. (2024). Alanyl-tRNA synthetase, AARS1, is a lactate sensor and lactyltransferase that lactylates p53 and contributes to tumorigenesis. Cell.

[26] Moreno-Yruela C., Zhang D., Wei W., Bæk M., Liu W., Gao J., Danková D., Nielsen A.L., Bolding J.E., Yang L. (2022). Class I histone deacetylases (HDAC1–3) are histone lysine delactylases. Sci. Adv..

[27] Hu X., Huang X., Yang Y., Sun Y., Zhao Y., Zhang Z., Qiu D., Wu Y., Wu G., Lei L. (2024). Dux activates metabolism-lactylation-MET network during early iPSC reprogramming with Brg1 as the histone lactylation reader. Nucleic Acids Res..

[28] Nuñez R., Sidlowski P.F., Steen E.A., Wynia-Smith S.L., Sprague D.J., Keyes R.F., Smith B.C. (2024). The TRIM33 bromodomain recognizes histone lysine lactylation. ACS Chem. Biol..

[29] Zhai G., Niu Z., Jiang Z., Zhao F., Wang S., Chen C., Zheng W., Wang A., Zang Y., Han Y. (2024). DPF2 reads histone lactylation to drive transcription and tumorigenesis. Proc. Natl. Acad. Sci. USA.

[30] Li S., Xu C., Fu Y., Lei P.-J., Yao Y., Yang W., Zhang Y., Washburn M.P., Florens L., Jaiswal M. (2018). DYRK1A interacts with histone acetyl transferase p300 and CBP and localizes to enhancers. Nucleic Acids Res..

[31] Kikuchi M., Morita S., Wakamori M., Sato S., Uchikubo-Kamo T., Suzuki T., Dohmae N., Shirouzu M., Umehara T. (2023). Epigenetic mechanisms to propagate histone acetylation by p300/CBP. Nat. Commun..

[32] Fan M., Yang K., Wang X., Chen L., Gill P.S., Ha T., Liu L., Lewis N.H., Williams D.L., Li C. (2023). Lactate promotes endothelial-to-mesenchymal transition via Snail1 lactylation after myocardial infarction. Sci. Adv..

[33] Porter N.J., Christianson D.W. (2019). Structure, mechanism, and inhibition of the zinc-dependent histone deacetylases. Curr. Opin. Struct. Biol..

[34] Latham T., Mackay L., Sproul D., Karim M., Culley J., Harrison D.J., Hayward L., Langridge-Smith P., Gilbert N., Ramsahoye B.H. (2012). Lactate, a product of glycolytic metabolism, inhibits histone deacetylase activity and promotes changes in gene expression. Nucleic Acids Res..

[35] Miranda-Gonçalves V., Lameirinhas A., Macedo-Silva C., Lobo J.C., Dias P., Ferreira V., Henrique R., Jerónimo C. (2020). Lactate increases renal cell carcinoma aggressiveness through sirtuin 1-dependent epithelial mesenchymal transition axis regulation. Cells.

[36] Levine D.C., Kuo H.-Y., Hong H.-K., Cedernaes J., Hepler C., Wright A.G., Sommars M.A., Kobayashi Y., Marcheva B., Gao P. (2021). NADH inhibition of SIRT1 links energy state to transcription during time-restricted feeding. Nat. Metab..

[37] Huang J., Wang X., Zhu Y., Li Z., Zhu Y.T., Wu J.C., Qin Z.H., Xiang M., Lin F. (2019). Exercise activates lysosomal function in the brain through AMPK-SIRT1-TFEB pathway. CNS Neurosci. Ther..

[38] Zhang C., Zhou T., Li C., Wang D., Tao J., Zhu X., Lu J., Ni J., Yao Y.-F. (2025). Deciphering novel enzymatic and non-enzymatic lysine lactylation in Salmonella. Emerg. Microbes Infect..

[39] Kong L.R., Gupta K., Wu A.J., Perera D., Ivanyi-Nagy R., Ahmed S.M., Tan T.Z., Tan S.L.-W., Fuddin A., Sundaramoorthy E. (2024). A glycolytic metabolite bypasses “two-hit” tumor suppression by BRCA2. Cell.

[40] Gaffney D.O., Jennings E.Q., Anderson C.C., Marentette J.O., Shi T., Oxvig A.-M.S., Streeter M.D., Johannsen M., Spiegel D.A., Chapman E. (2020). Non-enzymatic lysine lactoylation of glycolytic enzymes. Cell Chem. Biol..

[41] Henderson G.C., Horning M.A., Wallis G.A., Brooks G.A. (2007). Pyruvate metabolism in working human skeletal muscle. Am. J. Physiol.-Endocrinol. Metab..

[42] Liu X., Li S., Cui Q., Guo B., Ding W., Liu J., Quan L., Li X., Xie P., Jin L. (2024). Activation of GPR81 by lactate drives tumour-induced cachexia. Nat. Metab..

[43] Chen G., Liu J., Guo Y., Sun P. (2025). Mechanisms for Regulatory Effects of Exercise on Metabolic Diseases from the Lactate–Lactylation Perspective. Int. J. Mol. Sci..

[44] Wang Z., Xing T., Zhang L., Zhao L., Gao F. (2024). Dynamic changes of protein lactylation and their correlations with the glycolytic process during the postmortem acidification of broiler breast. Poult. Sci..

[45] Liu X., Xu Y., Zhao X., Bai Y., Ren C., Li X., Hou C., Zhang D. (2025). The role of lactate in meat beyond pH regulation: A study on lactylation and its effects on meat metabolism. Food Chem..

[46] Essén-Gustavsson B., Henriksson J. (1984). Enzyme levels in pools of microdissected human muscle fibres of identified type: Adaptive response to exercise. Acta Physiol. Scand..

[47] Kobayashi M. (2004). Fiber type-specific localization of monocarboxylate transporters MCT1 and MCT4 in rat skeletal muscle. Kurume Med. J..

[48] Vøillestad N., Tabata I., Medbø J. (1992). Glycogen breakdown in different human muscle fibre types during exhaustive exercise of short duration. Acta Physiol. Scand..

[49] Mattingly M.L., Anglin D.A., Ruple B.A., Scarpelli M.C., Bergamasco J.G., Godwin J.S., Mobley C.B., Frugé A.D., Libardi C.A., Roberts M.D. (2024). Acute and Chronic Resistance Training, Acute Endurance Exercise, nor Physiologically Plausible Lactate In Vitro Affect Skeletal Muscle Lactylation. Int. J. Mol. Sci..

[50] Chang J., Wu W., Qian P., Lu Z., He X., Wang F., Zhang T. (2025). Multi-omics study on the effect of moderate-intensity exercise on protein lactylation in mouse muscle tissue. Front. Cell Dev. Biol..

[51] Dai W., Wu G., Liu K., Chen Q., Tao J., Liu H., Shen M. (2023). Lactate promotes myogenesis via activating H3K9 lactylation-dependent up-regulation of Neu2 expression. J. Cachexia Sarcopenia Muscle.

[52] Desgeorges T., Galle E., Zhang J., von Meyenn F., De Bock K. (2024). Histone lactylation in macrophages is predictive for gene expression changes during ischemia induced-muscle regeneration. Mol. Metab..

[53] Sun W., Jia M., Feng Y., Cheng X. (2023). Lactate is a bridge linking glycolysis and autophagy through lactylation. Autophagy.

[54] Chen J., Zhang M., Liu Y., Zhao S., Wang Y., Wang M., Niu W., Jin F., Li Z. (2022). Histone lactylation driven by mROS-mediated glycolytic shift promotes hypoxic pulmonary hypertension. J. Mol. Cell Biol..

[55] Chen A., Chen Z., Huang B., Lian G., Luo L., Xie L. (2025). Hypoxia-induced histone lactylation promotes pulmonary arterial smooth muscle cells proliferation in pulmonary hypertension. Mol. Cell Biol..

[56] Jiang L., Zhyvylo I., Goncharov D., Teos L., Lin D., Franzi L., Saiyed A., Neeli S., Kenyon N., Wolters P. (2023). LDHA-Lactate Promotes Smooth Muscle Remodeling and Pulmonary Hypertension Through Lactylation of TOP1 and EMILIN1. Circulation.

[57] Zhu Y., Chen J.-C., Zhang J.-L., Wang F.-F., Liu R.-P. (2025). A new mechanism of arterial calcification in diabetes: Interaction between H3K18 lactylation and CHI3L1. Clin. Sci..

[58] Ma W., Jia K., Cheng H., Xu H., Li Z., Zhang H., Xie H., Sun H., Yi L., Chen Z. (2024). Orphan nuclear receptor NR4A3 promotes vascular calcification via histone lactylation. Circ. Res..

[59] Li X., Chen M., Chen X., He X., Li X., Wei H., Tan Y., Min J., Azam T., Xue M. (2024). TRAP1 drives smooth muscle cell senescence and promotes atherosclerosis via HDAC3-primed histone H4 lysine 12 lactylation. Eur. Heart J..

[60] Xu X., Zhang D.-D., Kong P., Gao Y.-K., Huang X.-F., Song Y., Zhang W.-D., Guo R.-J., Li C.-L., Chen B.-W. (2023). Sox10 escalates vascular inflammation by mediating vascular smooth muscle cell transdifferentiation and pyroptosis in neointimal hyperplasia. Cell Rep..

[61] Zhang Y., Jiang H., Dong M., Min J., He X., Tan Y., Liu F., Chen M., Chen X., Yin Q. (2024). Macrophage MCT4 inhibition activates reparative genes and protects from atherosclerosis by histone H3 lysine 18 lactylation. Cell Rep..

[62] Zhang J., Luo M.-Y., Li N.-P., Liang N., Yang Y.-H., Zhang Y.-R., Tan G.-K., Xie T., Gong S.-X., Wang A.-P. (2025). Histone lactylation-derived prelamin A accelerates pulmonary arterial smooth muscle cells senescence in hypoxia-induced pulmonary hypertension rats. Int. Immunopharmacol..

[63] Zhu X., Pang X., Wang X., Guan X., Tang Y., Wang Z., Zhang L., Zheng X., Li F., Mei J. (2025). Super-Enhancer–Driven LncRNA UNC5B-AS1 Inhibits Inflammatory Phenotypic Transition in Pulmonary Artery Smooth Muscle Cells via Lactylation. Arterioscler. Thromb. Vasc. Biol..

[64] Zhang T., Zhu Y., Wang X., Chong D., Wang H., Bu D., Zhao M., Fang L., Li C. (2024). The characterization of protein lactylation in relation to cardiac metabolic reprogramming in neonatal mouse hearts. J. Genet. Genom..

[65] Li R., Wang J., Zhao J., Liu J., Qin Y., Wang Y., Yuan Y., Kang N., Yao L., Yang F. (2025). Altered Lactylation Myocardial Tissue May Contribute to a More Severe Energy-Deprived State of the Tissue and Left Ventricular Outflow Tract Obstruction in HOCM. Bioengineering.

[66] Zhang T.-N., Huang X.-M., Li L., Li Y., Liu Y.-P., Shi X.-L., Wu Q.-J., Wen R., Yang Y.-H., Zhang T. (2025). Lactylation of HADHA promotes sepsis-induced myocardial depression. Circ. Res..

[67] She H., Hu Y., Zhao G., Du Y., Wu Y., Chen W., Li Y., Wang Y., Tan L., Zhou Y. (2024). Dexmedetomidine Ameliorates Myocardial Ischemia-Reperfusion Injury by Inhibiting MDH2 Lactylation via Regulating Metabolic Reprogramming. Adv. Sci..

[68] Luan X., Du R., Su G., Yan C., Ren X., Ju K., Jin Y., An Y., Guo D., Tian Z. (2025). Epigenetic regulation of cardiac tissue development by lysine lactylation. hLife.

[69] Zhang H., Feng Z., Tang K., Zhang L., Qiu Z., Qian L. (2025). 12-HEPE promotes cardiomyocyte proliferation by activating glycolysis and histone lactylation via Hippo signaling. Eur. J. Pharmacol..

[70] Ma X.M., Geng K., Wang P., Jiang Z., Law B.Y.-K., Xu Y. (2024). MCT4-dependent lactate transport: A novel mechanism for cardiac energy metabolism injury and inflammation in type 2 diabetes mellitus. Cardiovasc. Diabetol..

[71] Wang N., Wang W., Wang X., Mang G., Chen J., Yan X., Tong Z., Yang Q., Wang M., Chen L. (2022). Histone lactylation boosts reparative gene activation post–myocardial infarction. Circ. Res..

[72] Yu W., Kong Q., Jiang S., Li Y., Wang Z., Mao Q., Zhang X., Liu Q., Zhang P., Li Y. (2024). HSPA12A maintains aerobic glycolytic homeostasis and Histone3 lactylation in cardiomyocytes to attenuate myocardial ischemia/reperfusion injury. JCI Insight.

[73] Wang L., Li D., Yao F., Feng S., Tong C., Rao R., Zhong M., Wang X., Feng W., Hu Z. (2025). Serpina3k lactylation protects from cardiac ischemia reperfusion injury. Nat. Commun..

[74] Lv J., Yin M., Jin H. (2025). Hypoxia Aggravates Myocardial Ischemia/Reperfusion Injury Through the Promotion of Ferroptosis via ACSL4 Lactylation. J. Cardiovasc. Transl. Res..

[75] Wang Y., Yue Q., Song X., Du W., Liu R. (2025). Hypoxia/reoxygenation-induced Glycolysis Mediates Myocardial Ischemia–reperfusion Injury Through Promoting the Lactylation of GPX4. J. Cardiovasc. Transl. Res..

[76] Fang L., Yu Z., Qian X., Fang H., Wang Y. (2024). LDHA exacerbates myocardial ischemia-reperfusion injury through inducing NLRP3 lactylation. BMC Cardiovasc. Disord..

[77] Xu G.-E., Yu P., Hu Y., Wan W., Shen K., Cui X., Wang J., Wang T., Cui C., Chatterjee E. (2024). Exercise training decreases lactylation and prevents myocardial ischemia–reperfusion injury by inhibiting YTHDF2. Basic Res. Cardiol..

[78] Zhang N., Zhang Y., Xu J., Wang P., Wu B., Lu S., Lu X., You S., Huang X., Li M. (2023). α-myosin heavy chain lactylation maintains sarcomeric structure and function and alleviates the development of heart failure. Cell Res..

[79] Zhao S.S., Liu J., Wu Q.C., Zhou X.L. (2024). Lactate regulates pathological cardiac hypertrophy via histone lactylation modification. J. Cell. Mol. Med..

[80] Wang B., Ma J., Yang D. (2025). Role of PFKM lactylation in glycolysis regulation in endometrial cancer cells. Genes Dis..

[81] Wang J., Yang P., Yu T., Gao M., Liu D., Zhang J., Lu C., Chen X., Zhang X., Liu Y. (2022). Lactylation of PKM2 suppresses inflammatory metabolic adaptation in pro-inflammatory macrophages. Int. J. Biol. Sci..

[82] Pan R.-Y., He L., Zhang J., Liu X., Liao Y., Gao J., Liao Y., Yan Y., Li Q., Zhou X. (2022). Positive feedback regulation of microglial glucose metabolism by histone H4 lysine 12 lactylation in Alzheimer’s disease. Cell Metab..

[83] Chen X., Huang W., Zhang J., Li Y., Xing Z., Guo L., Jiang H., Zhang J. (2023). High-intensity interval training induces lactylation of fatty acid synthase to inhibit lipid synthesis. BMC Biol..

[84] Wang Y., Sun J., Xue L., Sun Y., Zhang K., Fan M., Qian H., Li Y., Wang L. (2025). Chlorogenic Acid Improves High-Fat Diet-Induced Skeletal Muscle Metabolic Disorders by Regulating Mitochondrial Function and Lactate Metabolism. J. Agric. Food Chem..

[85] Wang Y., Wang F., Sun J., Xue L., Sun Y., Zhang K., Fan M., Qian H., Yang B., Du J. (2025). Gallic Acid Ameliorates Skeletal Muscle Metabolic Inflexibility by Regulating Lactate Metabolism and Promoting Mitochondrial Function. Mol. Nutr. Food Res..

[86] Zhao Q. (2017). On the indirect relationship between protein dynamics and enzyme activity. Prog. Biophys. Mol. Biol..

[87] Mattingly M.L., Ruple B.A., Sexton C.L., Godwin J.S., McIntosh M.C., Smith M.A., Plotkin D.L., Michel J.M., Anglin D.A., Kontos N.J. (2023). Resistance training in humans and mechanical overload in rodents do not elevate muscle protein lactylation. Front. Physiol..

[88] Nowacka A., Śniegocki M., Śniegocka M., Ziółkowska E.A. (2025). Nicotinamide and Pyridoxine in Muscle Aging: Nutritional Regulation of Redox, Inflammation, and Regeneration. Antioxidants.

[89] Torres-Méndez J.K., Niño-Narvión J., Martinez-Santos P., Diarte-Añazco E.M.G., Méndez-Lara K.A., Del Olmo T.V., Rotllan N., Julián M.T., Alonso N., Mauricio D. (2023). Nicotinamide Prevents Diabetic Brain Inflammation via NAD+-Dependent Deacetylation Mechanisms. Nutrients.

[90] Zhang X., Meng F., Lyu W., He J., Wei R., Du Z., Zhang C., Guan Y., Huang X., Lyu G. (2023). Histone lactylation antagonizes senescence and skeletal muscle aging via facilitating gene expression reprogramming. bioRxiv.

[91] Zou K., Hinkley J.M., Park S., Zheng D., Jones T.E., Pories W.J., Hornby P.J., Lenhard J., Dohm G.L., Houmard J.A. (2019). Altered tricarboxylic acid cycle flux in primary myotubes from severely obese humans. Int. J. Obes..

[92] Zhou R., Ding R.-C., Yu Q., Qiu C.-Z., Zhang H.-Y., Yin Z.-J., Ren D.-L. (2024). Metformin attenuates neutrophil recruitment through the H3K18 lactylation/reactive oxygen species pathway in zebrafish. Antioxidants.

[93] Xu R., Yuan W., Wang Z. (2023). Advances in glycolysis metabolism of atherosclerosis. J. Cardiovasc. Transl. Res..

[94] Grootaert M.O., Moulis M., Roth L., Martinet W., Vindis C., Bennett M.R., De Meyer G.R. (2018). Vascular smooth muscle cell death, autophagy and senescence in atherosclerosis. Cardiovasc. Res..

[95] Ouyang J., Wang H., Huang J. (2023). The role of lactate in cardiovascular diseases. Cell Commun. Signal..

[96] Tsao C.W., Aday A.W., Almarzooq Z.I., Alonso A., Beaton A.Z., Bittencourt M.S., Boehme A.K., Buxton A.E., Carson A.P., Commodore-Mensah Y. (2022). Heart disease and stroke statistics—2022 update: A report from the American Heart Association. Circulation.

[97] Bei Y., Zhu Y., Zhou J., Ai S., Yao J., Yin M., Hu M., Qi W., Spanos M., Li L. (2024). Inhibition of Hmbox1 promotes cardiomyocyte survival and glucose metabolism through Gck activation in ischemia/reperfusion injury. Circulation.

[98] Bosso G., Mercurio V., Diab N., Pagano A., Porta G., Allegorico E., Serra C., Guiotto G., Numis F.G., Tocchetti C.G. (2021). Time-weighted lactate as a predictor of adverse outcome in acute heart failure. ESC Heart Fail..

[99] Wang X., Fan W., Li N., Ma Y., Yao M., Wang G., He S., Li W., Tan J., Lu Q. (2023). YY1 lactylation in microglia promotes angiogenesis through transcription activation-mediated upregulation of FGF2. Genome Biol..

[100] Ferguson B.S., Rogatzki M.J., Goodwin M.L., Kane D.A., Rightmire Z., Gladden L.B. (2018). Lactate metabolism: Historical context, prior misinterpretations, and current understanding. Eur. J. Appl. Physiol..

